# Impact of the Coronavirus Pandemic on Patients Requiring Tracheal Intubation by Helicopter Emergency Medical Services: A Retrospective, Single-Center, Observational Study

**DOI:** 10.3390/jcm13133694

**Published:** 2024-06-25

**Authors:** Kentaro Hayashi, Jin Kikuchi, Hidekazu Hishinuma, Takafumi Noguchi, Masayoshi Zaitsu, Koji Wake

**Affiliations:** 1 Department of Emergency and Critical Care Medicine, Dokkyo Medical University, Tochigi 321-0293, Japan; k-jin@dokkyomed.ac.jp (J.K.); wake@dokkyomed.ac.jp (K.W.); 2 Data Science Center, Jichi Medical University, Tochigi 329-0498, Japan; 3 Department of Public Health, School of Medicine, Dokkyo Medical University, Tochigi 321-0293, Japan; h-hishi@dokkyomed.ac.jp; 4 Department of Adult Nursing, School of Nursing, Dokkyo Medical University, Tochigi 321-0293, Japan; noguchi1@dokkyomed.ac.jp; 5 Center for Research of the Aging Workforce, University of Occupational and Environmental Health, Fukuoka 807-8555, Japan; mzaitsu@med.uoeh-u.ac.jp

**Keywords:** COVID-19, helicopter emergency medical services, pre-hospital intervention, tracheal intubation

## Abstract

**Background/Objectives**: The impacts of the coronavirus disease 2019 (COVID-19) pandemic on patients using helicopter emergency medical services (HEMS) regarding tracheal intubation and patient management remain unclear. Thus, we aimed to investigate this matter in Japan. **Methods**: In this retrospective, observational study, we analyzed 2277 patients who utilized HEMS in Tochigi Prefecture during 2018–2022. We included only patients who required tracheal intubation. We categorized patients from February 2020 to January 2022 in the pandemic group and those from February 2018 to January 2020 in the control group. We compared the interval from arrival at the scene to leaving the scene (on-scene time) and secondary variables between the two groups. **Results**: A total of 278 eligible patients were divided into the pandemic group *(n* = 127) and the control group (*n* = 151). The on-scene time was lower during the pandemic than that before (25.64 ± 9.19 vs. 27.83 ± 8.74 min, *p* = 0.043). The percentage of patients using midazolam was lower (11.8% vs. 22.5%, *p* = 0.02) and that of patients using rocuronium bromide was higher (29.1% vs. 6.0%, *p* < 0.001) during the pandemic. In contrast, the type of intervention other than tracheal intubation and the type of transportation to the hospital did not differ between the groups. **Conclusions**: The COVID-19 pandemic was associated with changes in the mission time of and the frequency of certain drugs administered by the HEMS. However, the type of intervention and the type of transportation did not differ. Further research is needed on changes in patient prognosis and condition due to the effects of the COVID-19 pandemic.

## 1. Introduction

Helicopter emergency medical services (HEMS), initially launched in Germany and the U.S.A. in 1970, has now been adopted worldwide to meet the needs of patients with urgent and severe emergency conditions (e.g., trauma, toxicosis, and anaphylaxis) and endogenous emergencies (e.g., cardiovascular diseases) [1,2,3]. In Japan, HEMS was established in 2001, and Tochigi prefecture started HEMS in 2011 [4]. The number of patients using HEMS in Tochigi is approximately one thousand per year [4]. In this service, a helicopter ambulance called “Doctor-Heli” transports emergency physicians to provide immediate, on-scene, advanced medical treatment, including ultrasound examination, tracheal intubation, and pleural drainage, within 30 min [3].

The use of HEMS was partly limited by the coronavirus disease 2019 (COVID-19) pandemic. For instance, on 3 January 2020, the Japanese government recommended that people wear a mask, disinfect their hands, and avoid the three Cs, namely, closed spaces with poor ventilation, crowded spaces with many people, and close contact, including intimate conversation, loud cheering, and exercise within close proximity to other persons, to prevent severe acute respiratory syndrome coronavirus 2 (SARS-CoV-2) infection [5]. Following this policy, on 21 April 2020, the Japanese Society for Aeromedical Services (JSAS) recommended that physician-staffed HEMS not be used to transport patients with COVID-19 or those who possibly have COVID-19 according to medical tests performed by the HEMS staff [6].

Regarding the risk of exposure to COVID-19 among HEMS staff, tracheal intubation is known as the most hazardous aerosol-generating procedure [7]. In addition, although personal protective equipment (PPE) can protect HEMS staff from COVID-19 exposure during intubation, it complicates the procedure [7].

The impact of the COVID-19 pandemic on the activity of HEMS and the tracheal intubation they perform has not been fully investigated. Studies have suggested that the pandemic increased the infection risk among HEMS staff, necessitating that the number of personnel on board be minimized to mitigate exposure [8,9,10]. However, the impact of the pandemic on patients of HEMS regarding tracheal intubation and patient management, such as the on-scene operative time, remain unclear.

The aim of this study was to assess the potential impact of the COVID-19 pandemic on patients who required tracheal intubation during HEMS operations. Contrary to concerns about adverse impacts on HEMS activities, the on-scene time was not prolonged owing to adjustments and modifications of on-scene medication.

## 2. Materials and Methods

### 2.1. Study Design, Setting, Patient Selection

**Figure 1:**
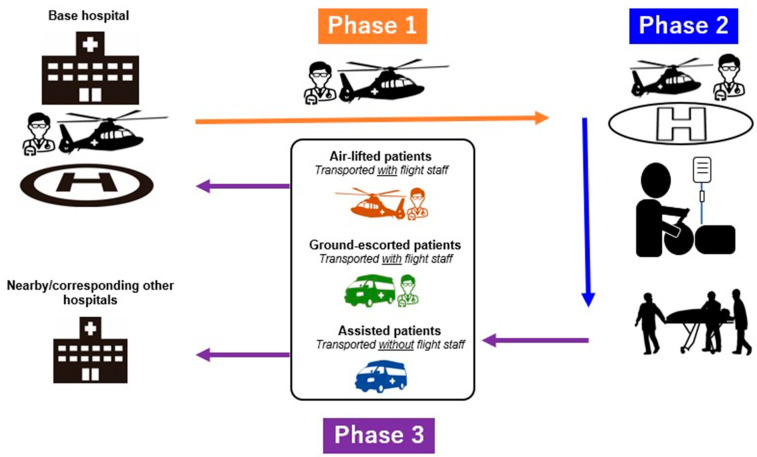
Phases of a Tochigi helicopter emergency medical services mission. Phase 1—dispatch, which includes the initial call, helicopter takeoff, and arrival at the mission scene. Phase 2—on-scene operations, encompassing activities from the helicopter’s arrival at the scene to its departure from the scene. Phase 3—patient transport, extending from departure from the scene to arrival at the most suitable designated hospital.

In this retrospective study, we analyzed the data of patients in Tochigi who were aided by HEMS and required tracheal intubation on mission sites (2018–2022). Information on Tochigi HEMS has been outlined in a previous study [4]. Briefly, Tochigi prefecture has 1.9 million people (1.5% of Japan’s total population), with an area of 6408 km^2^, stretching across approximately 84 km east–west and 98 km north–south. It is located 100 km to the north of Tokyo. The helicopter ambulance, which can reach anywhere in the prefecture within 20 min with onboard emergency physicians and nurses, is kept on standby at the base hospital. Each Tochigi HEMS mission comprises four distinct phases (Figure 1). Phase 1 comprises the dispatch, which includes the initial call, helicopter takeoff, and arrival at the mission scene. Phase 2 comprises on-scene operations, encompassing activities from the helicopter’s arrival at the scene to its departure from the scene. Phase 3 comprises patient transport, extending from departure from the scene to arrival at the most suitable designated hospital. In Phase 3, the onboard emergency physicians decide on the mode of transport of the patient to the base hospital or other hospitals. They decide whether to transport the patient via the HEMS or the automotive ambulance (ground emergency medical services, GEMS), and whether those who are transported via GEMS should be accompanied by emergency physicians [4].

We collected data of all patients who used HEMS during Tochigi HEMS dispatch missions from 1 February 2018 to 31 January 2022. We excluded transfer missions, cancelled missions, and missions for which the on-scene time was unknown, and we included patients who required tracheal intubation.

### 2.2. Main Exposure: COVID-19 Pandemic

The main exposure was the COVID-19 pandemic. All patients were categorized into the pandemic group (1 February 2020–31 January 2022) or the control group (1 February 2018–31 January 2020).

In the HEMS operations in Tochigi prefecture, we changed the response method in clinical settings based on the consensus of the WHO [11] and information on the virus. What changed during the COVID-19 pandemic were the mandatory equipment of PPE, including N95 masks for physicians in all operations regardless of tracheal intubation; the mandatory use of HEPA filtration systems [12] after tracheal intubation; and the management of doctors to reduce the generation of aerosols.

### 2.3. Patient Background and Other HEMS Operation Characteristics

The following background information and other characteristics were collected according to a previous study [4]: patient age and sex, number of physicians, on-scene diagnosis, pre-hospital severity, Glasgow Coma Scale score, blood pressure, heart rate, peripheral capillary oxygen saturation, body temperature, and respiratory rate.

### 2.4. Outcomes

We determined on-scene time, that is, the activity time during on-scene operations (phase 2), as the primary outcome. Phase 2 was considered to have the greatest impact on COVID-19 treatment, as that is when tracheal intubation is performed. The secondary outcomes were the drugs used for tracheal intubation (propofol, midazolam, rocuronium bromide, buprenorphine hydrochloride, and ketamine), on-scene interventions (intravenous drip, oxygen administration, ultrasound examination, pleural drainage, chest compression, and external defibrillation), and the types of transportation used for phase 3.

For reference, the on-scene time was calculated for phase 2 also for patients who were not intubated to verify whether COVID-19 affected on-scene time for such patients. For phase 3 and the total mission time, we limited the analysis to air-lifted patients who were transported to the base hospital via helicopter ambulance, because the times related to other transportation types were not recorded in the database.

### 2.5. Statistical Analysis

Data were compared using the *t*-test or the chi-square test with IBM SPSS Statistics for Windows version 27 (IBM Corp., Armonk, NY, USA). *p*-values < 0.05 were considered statistically significant. Data are presented as the frequency (%) or mean ± standard deviation.

The protocol of this study was approved by the institutional review board of Dokkyo Medical University Hospital, and examinations were conducted according to the standards of good clinical practice and the Helsinki Declaration.

## 3. Results

**Figure 2:**
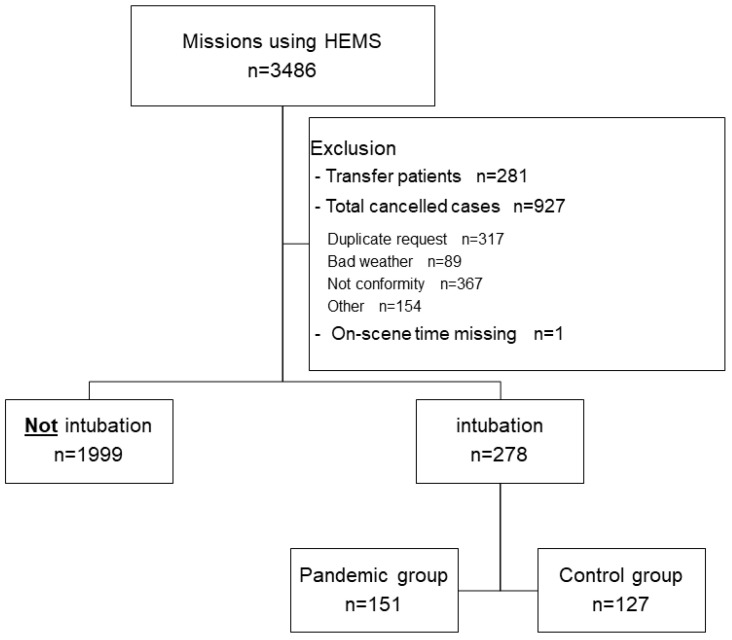
All the Tochigi HEMS missions and inclusion of patients. Of the 3486 patients using HEMS during 1 February 2018–31 January 2022, 278 were included in the analysis. Abbreviation: HEMS, helicopter emergency medical services.

Overall, 3486 HEMS missions were conducted during the study period, of which 1209 were excluded. Of the 2277 patients that remained, 278 required tracheal intubation and 1999 did not. Finally, the pandemic group comprised 127 patients, and the control group comprised 151 (Figure 2). Table 1 summarizes background characteristics, clinical characteristics, and the numbers of physicians on board, none of which differed between the groups.

**Figure 3:**
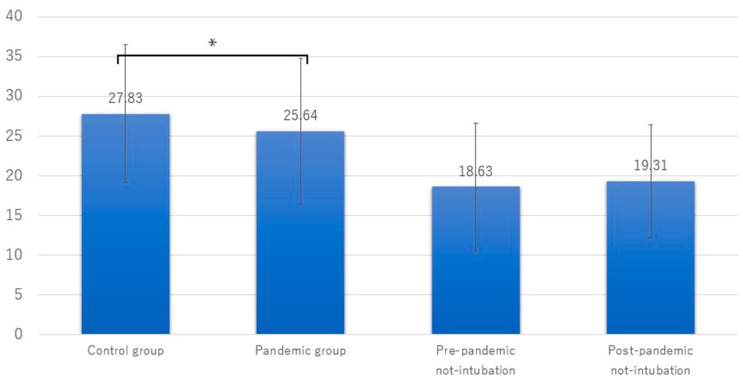
The bars represent the primary outcome, that is, on-scene time. The number in each bar represents the mean on-scene time in minutes. The asterisk indicates a significant difference, determined via the *t*-test.

Table 2 summarizes the outcomes and treatments at the mission sites between the two groups. The on-scene time significantly differed before and during the COVID-19 pandemic for patients who needed tracheal intubation, being lower during the pandemic (Figure 3). In contrast, the on-scene time was unchanged for patients who did not require tracheal intubation. The percentage of patients using midazolam was lower during the pandemic, whereas the percentage using rocuronium bromide was higher during the pandemic than before. The usage patterns for other medications did not differ between the two periods. The distributions of other interventions and the type of patient transportation from the mission site to the designated hospital also did not differ between the periods.

In a supplementary analysis restricted to patients transported to the base hospital via HEMS, no differences were observed except for the use of rocuronium bromide (Tables S1 and S2). In addition, we showed detailed numbers of total mission and the pandemic group by month (Figure S1), and showed the overview of viral waves [13].

## 4. Discussion

In the present study, we aimed to determine the impact of the COVID-19 pandemic on patients who required tracheal intubation during HEMS operations in Tochigi prefecture. To date, few studies have been conducted on changes in HEMS before and during the pandemic, and this was, to our knowledge, the first to focus on such patients requiring tracheal intubation. We hypothesized that the impact of COVID-19 and the risk of infection would have adverse effects on HEMS, such as an extension of the on-scene time. However, the on-scene time remained unchanged and even decreased in certain groups.

Exposure to infected aerosols from intubated patients is a major concern for healthcare providers. Recent studies confirmed the infectious risks posed by aerosols generated from patients with COVID-19 during intubation and artificial ventilation [14,15,16]. These studies highlighted the importance of infection control measures to minimize the risk of aerosol generation and the risk of infection. The requirement of infection control measures, such as the use of PPE, might raise concerns about increasing on-scene time. Nevertheless, the results of the present study demonstrate that such measures did not have a negative impact on on-scene time. In order to protect the safety of healthcare providers, the actions taken to suppress the patient’s cough reflex and prevent exposure to COVID-19 resulted in a reduction in on-scene time, which was beneficial for the patient. The present study is also considered to be a very valuable social experiment result in terms of the nudge effect in behavioral economics.

The favorable outcomes regarding on-scene time in this study may be partly attributed to the increased frequency of rocuronium bromide use for tracheal intubation. Neuromuscular blocking agents (NMBAs) offer optimal intubation conditions and shorten the procedural time [17,18]. This advantage became more pronounced during the COVID-19 pandemic, given the higher demand for endotracheal intubation of patients critically ill with COVID-19 [19,20]. This trend was also observed in our study. Similarly, the frequency of midazolam use may have decreased and that of propofol use may have increased as the result of the physician’s consideration to prevent the occurrence of aerosols and coughs [21]. Thus, the on-scene time was not extended, and even decreased, owing to adjustments and modifications of on-scene medication. Based on these results, we recommend that HEMS use NMBAs and deep sedation during tracheal intubation of potentially infectious patients for the safety of healthcare professionals and to decrease the on-scene time.

Several studies [22,23,24,25] have demonstrated that the presence of physicians in HEMS significantly improves survival rates of patients who have experienced physical trauma, primarily owing to prompt procedures, such as tracheal intubation and chest drainage, along with rapid fluid infusion. These on-site treatments are considered critical in reducing mortality rates and are more effective in HEMS than those in traditional ambulance services. In the present study, the proportions of field procedures, such as tracheal intubation and chest drainage, did not differ before and during the pandemic and could not have a confounding effect on survival.

In the present study, the number of HEMS transportations during phase 3 decreased, whereas the number of GEMS transportations increased, although this change was not significant. The choice of transportation is a crucial contributor to the total mission time. When physicians encounter patients likely to be a high infection risk, they may prioritize the safety of the crew, especially non-healthcare professionals such as pilots and mechanics, and change the transportation method to a traditional ambulance. However, contrary to our expectations, this scenario seemed uncommon in Tochigi prefecture during the pandemic.

In terms of SARS-CoV-2 infection, tracheal intubation in HEMS operations may carry a high risk of complications, and several factors may increase the difficulty of and risk associated with the procedure. Although the interventions and type of transport were not significantly affected by the pandemic, the frequency of rocuronium bromide and propofol use during tracheal intubation significantly increased and the frequency of midazolam decreased, and the on-scene time decreased. Crew members need to receive adequate training and take appropriate precautions to mitigate these risks and ensure the safety of both themselves and the patients.

In a previous study [26], the number of HEMS operations decreased during the COVID-19 pandemic. HEMS dispatches were not requested by the fire department when they encountered signs of infection, such as fever, dyspnea, or a low oxygen saturation value, as our hospital decided not to transport patients with possible COVID-19 infection after receiving the announcement from the JSAS [6]. Dispatches for other diseases during the COVID-19 pandemic might also have decreased because people were staying home and, as a result, the number of traffic accidents or occupational accidents might have decreased [9]. The COVID-19 pandemic was generally associated with an overall absolute reduction in road traffic collisions and related deaths and injuries, despite the relative increase in accident severity [27,28]. The present study revealed that the overall number of missions decreased regardless of the use of tracheal intubation, the number of patients who needed tracheal intubation during the COVID-19 pandemic did not differ significantly from before, and the clinical characteristics (age, sex, pre-hospital severity, diagnosis, and vital signs) were not affected. This suggests that patients with severe conditions, such as those who required HEMS or tracheal intubation, were properly managed in the study population.

This study has several limitations. First, it was a retrospective analysis of real-world HEMS data of a single prefecture, relying on the decisions of individual physicians, which might have introduced a bias in decisions and limited the generalizability of the results. Nonetheless, Tochigi HEMS maintain a high quality of medical treatment and follow standard procedures [4]. Second, the severity of each disease was subjectively determined by physicians in prehospital settings, potentially affecting the accuracy of their assessments. Additionally, provisional diagnoses made at the site might have differed from definitive diagnoses established at the hospital, which we did not verify in this study. Third, we did not assess the safety of tracheal intubations in HEMS in this study owing to insufficient data availability. Specifically, details such as the number of intubation attempts and the devices used for intubation were not recorded in the dataset [8,9,10,29,30]. Fourth, we did not describe changes in the response method after the request of HEMS, as alterations in emergency request protocols, current laws, and HEMS selection criteria might have occurred before and during the COVID-19 pandemic. However, the characteristics of patients who were intubated did not differ before and during the pandemic.

## 5. Conclusions

To our knowledge, this is the first documentation of the potential impact of the COVID-19 pandemic on patients who required tracheal intubation from HEMS in one prefecture in Japan. Contrary to concerns about adverse impacts on HEMS activities, on-scene time was not prolonged owing to adjustments and modifications of on-scene medication. Further research using both local- and national-level data is needed to accelerate our understanding of the changes in patient prognosis and condition because of COVID-19.

## Figures and Tables

**Table 1:** Clinical characteristics and numbers of physicians of patients with HEMS requiring tracheal intubation before and during the COVID-19 pandemic.

	Total	Control Group	Pandemic Group	*p*-Value
	N = 278	n = 151	n = 127	
Age (years), mean ± SD	61.05 ± 20.23	60.46 ± 20.80	61.76 ± 19.58	0.595
Sex (male), n (%)	189 (68.0)	103 (68.2)	86 (67.7)	0.930
Number of physicians (mean ± SD)	1.53 ± 0.50	1.50 ± 0.50	1.57 ± 0.50	0.244
Pre-hospital severity, n (%)				0.831
Mild	0 (0.0)	0 (0.0)	0 (0.0)	
Moderate	16 (5.8)	9 (6.0)	7 (5.5)	
Severe	258 (92.8)	140 (92.7)	118 (92.9)	
Death	2 (0.7)	1 (0.7)	1 (0.8)	
Missing	2 (0.7)	1 (0.7)	1 (0.8)	
Diagnosis, n (%)				0.808
Trauma	82 (29.5)	48 (31.8)	34 (26.8)	
Neurology	24 (8.6)	16 (10.6)	8 (6.3)	
Cardiovascular disease				
Ischemic heart disease	10 (3.6)	7 (4.6)	3 (2.4)	
Aortic disease	9 (3.2)	4 (2.6)	5 (3.9)	
Stroke	63 (22.7)	31 (20.5)	32 (25.2)	
Others	8 (2.9)	4 (2.6)	4 (3.1)	
Cardiopulmonary arrest	54 (19.4)	26 (17.2)	28 (22)	
Respiratory disease	5 (1.8)	3 (2.0)	2 (1.6)	
Gastroenterology	0 (0.0)	0 (0.0)	0 (0.0)	
Allergies	2 (0.7)	1 (0.7)	1 (0.8)	
Toxicosis	4 (1.4)	3 (2.0)	1 (0.8)	
Other disease	17 (6.1)	8 (5.3)	9 (7.1)	
Vital signs				
GCS score, n (%)				0.143
3–8	230 (82.7)	119 (78.8)	111 (87.4)	
9–13	29 (10.4)	19 (12.6)	10 (7.9)	
14–15	17 (6.1)	12 (7.9)	5 (3.9)	
Missing	2 (0.7)	1 (0.7)	1 (0.8)	
BP < 80 mmHg, n (%)	63 (22.7)	40 (26.5)	23 (18.1)	0.096
Missing	0 (0.0)	0 (0.0)	0 (0.0)	
HR > 130 bpm, n (%)	62 (22.3)	27 (17.9)	35 (27.6)	0.053
Missing	0 (0.0)	0 (0.0)	0 (0.0)	
SpO_2_ < 80%, n (%)	7 (2.5)	3 (2.0)	4 (3.1)	0.613
Missing	35 (12.6)	24 (15.9)	11 (8.7)	
RR > 35 or <8 breaths/min, n (%)	106 (38.1)	59 (39.1)	47 (37.0)	0.724
Missing	0 (0.0)	0 (0.0)	0 (0.0)	
O_2_ administration, n (%)	246 (88.5)	134 (88.7)	112 (88.2)	0.622
Missing	13 (4.7)	6 (4.0)	7 (5.5)	
BT (°C), mean ± SD	36.67 ± 2.25	36.67 ± 2.29	36.67 ± 2.24	0.997
Missing	131 (47.1)	86 (57.0)	45 (35.4)	

Abbreviations: BP, blood pressure; BT, body temperature; COVID-19, coronavirus disease 2019; GCS, Glasgow Coma Scale; HEMS, helicopter emergency medical services; HR, heart rate; RR, respiratory rate; SD, standard deviation; SpO_2_, oxygen saturation.

**Table 2:** Outcomes of patients who required tracheal intubation before and during the COVID-19 pandemic.

	Total	Control	Pandemic	*p*-Value
	n = 278	n = 151	n = 127	
Interval (min), mean ± SD				
Phase 2 (on-scene time)	26.83 ± 9.00	27.83 ± 8.74	25.64 ± 9.19	0.043
Drugs used, n (%)				
Propofol	124 (44.6)	65 (43)	59 (46.5)	0.569
Midazolam	49 (17.6)	34 (22.5)	15 (11.8)	0.020
Rocuronium bromide	46 (16.5)	9 (6.0)	37 (29.1)	<0.001
Buprenorphine hydrochloride	10 (3.6)	6 (4.0)	4 (3.1)	0.759
Ketamine	18 (6.5)	12 (7.9)	6 (4.7)	0.277
Other interventions, n (%)				
Intravenous drip	270 (97.1)	149 (98.7)	121 (95.3)	0.148
Oxygen administration	249 (89.6)	139 (92.1)	110 (86.6)	0.139
Ultrasound examination	192 (69.1)	109 (72.2)	83 (65.4)	0.220
Pleural drainage	13 (4.7)	8 (5.3)	5 (3.9)	0.777
Chest compression	42 (15.1)	19 (12.6)	23 (18.1)	0.200
External defibrillation	38 (13.7)	21 (13.9)	17 (13.4)	0.900
Type of transportation, n (%)				0.056
To the base hospital by HEMS with physician	152 (54.7)	82 (54.3)	70 (55.1)	
To the base hospital by GEMS with physician	15 (5.4)	3 (2.0)	12 (9.4)	
To another hospital by HEMS with physician	91 (32.7)	56 (37.1)	35 (27.6)	
To another hospital by GEMS with physician	14 (5.0)	7 (4.6)	7 (5.5)	
To another hospital by GEMS without physician	5 (1.8)	3 (2.0)	2 (1.6)	
Missing	1 (0.4)	0 (0.0)	1 (0.8)	

Abbreviations: COVID-19, coronavirus disease 2019; GEMS, ground emergency medical services; HEMS, helicopter emergency medical services; SD, standard deviation.

## Data Availability

The datasets used and/or analyzed during the current study are available from Dr. Kentaro Hayashi on reasonable request. Restrictions apply to the availability of these data, which were used under license for this study and so are not publicly available.

## Supplementary Materials

The following supporting information can be downloaded at: https://www.mdpi.com/article/10.3390/jcm13133694/s1, Table S1: Background and characteristics, restricted to patients transported to the base hospital by HEMS, accompanied by a physician; Table S2: Outcomes, restricted to patients transported to the base hospital by HEMS, accompanied by a physician; Figure S1: The number of total missions and the number of target cases (intubation) for each month, and the duration of each viral wave in Japan’s announcement [11]: 1st wave (Week 13 of 2020~Week 20 of 2020), 2nd wave (26th week 2020~39th week 2020), 3rd wave (44th week 2020~8th week 2021), 4th wave; alpha (2021 week 9~2021 week 24), 5th wave; delta (2021 week 28~2021 week 38), 6th wave; omicron (Week 51 of 2021~Week 24 of 2022).
