# Supplementary figures and images for: Impact of the Coronavirus Pandemic on Patients Requiring Tracheal Intubation by Helicopter Emergency Medical Services: A Retrospective, Single-Center, Observational Study
Source: J Clin Med. 2024 Jun 25;13(13):3694. doi: 10.3390/jcm13133694 (PMC11242781; doi:10.3390/jcm13133694)

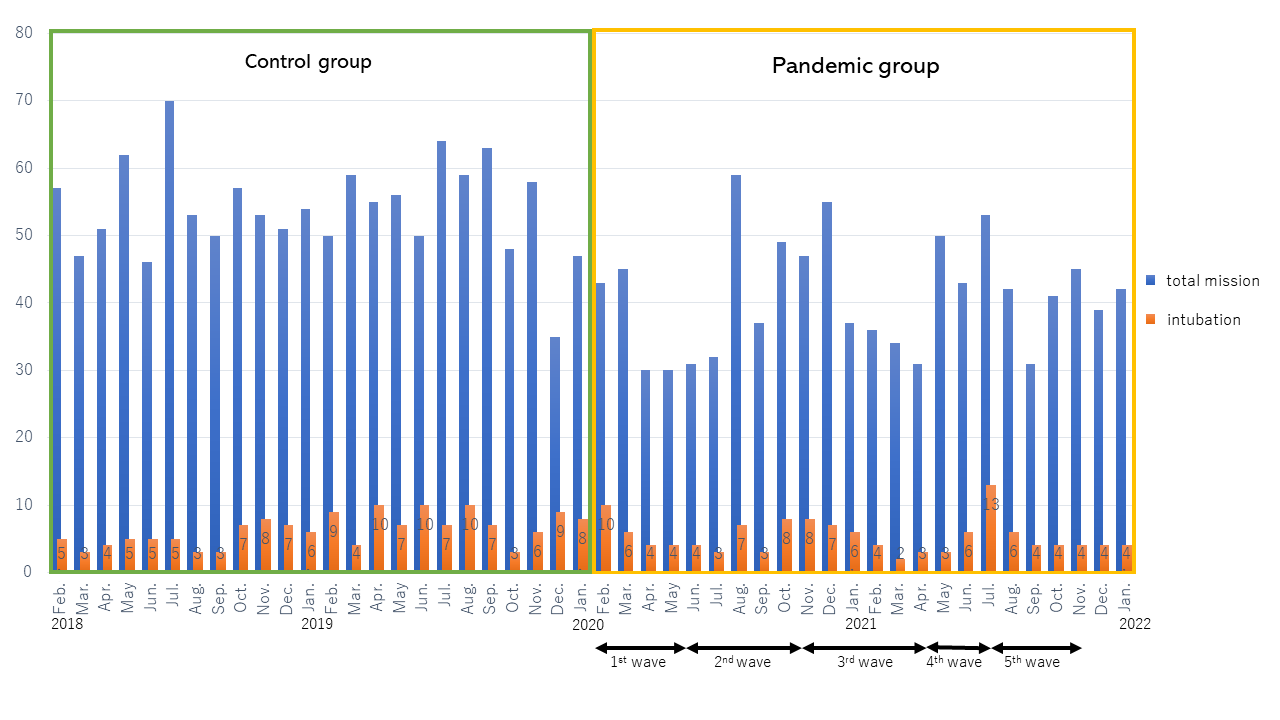

Supplement: Supplementary file 1 [file jcm-13-03694-s001.zip › Supplemental/Figure S1.tif]
